# Long-Term Clinical Outcomes of Ulcerative Colitis with Concurrent Endoscopic and Histologic Remission

**DOI:** 10.3390/medicina61111968

**Published:** 2025-11-02

**Authors:** Ji Min Lee, Kang-Moon Lee, Dae Bum Kim, Ji-Han Jung

**Affiliations:** 1 Department of Internal Medicine, St. Vincent’s Hospital, College of Medicine, The Catholic University of Korea, Seoul 16247, Republic of Korea; yulialee@naver.com (J.M.L.);; 2 Department of Hospital Pathology, St. Vincent’s Hospital, College of Medicine, The Catholic University of Korea, Seoul 16247, Republic of Korea

**Keywords:** ulcerative colitis, histologic remission, relapse

## Abstract

*Background and Objectives*: Therapeutic goals for ulcerative colitis (UC) have expanded beyond symptom control to include mucosal and histological healing. However, the long-term prognostic value of achieving both targets remains uncertain, particularly in Asian populations. This study aimed to evaluate long-term outcomes and relapse predictors in patients with UC who achieved both endoscopic and histologic remission. *Materials and Methods*: This prospective observational study consecutively enrolled adults with clinically inactive UC who attained endoscopic remission (Mayo endoscopic subscore = 0) and histologic remission (Nancy index ≤1) between June 2014 and May 2018. Demographic, clinical, and laboratory data—including fecal calprotectin—were collected. Clinical relapse was defined as a Mayo score increase >3 or initiation of systemic corticosteroids or biologics. Patients were followed longitudinally for a median of 55 months (minimum 12 months), and relapse risk was evaluated using Kaplan–Meier and univariate Cox regression analyses. *Results*: A total of 41 patients were included (mean age 54 ± 14 years; 56% male). The median follow-up was 54 months (range 17–78). Ten patients (24.4%) relapsed during follow-up, with cumulative relapse rates of 9.8%, 10.3%, 15.8%, and 24.1% at 12, 24, 36, and 48 months, respectively. Kaplan–Meier analyses demonstrated significantly higher relapse in patients with non-E1 disease (E2 + E3, *p* = 0.021), immunomodulator use (*p* = 0.008), and biologics use (*p* = 0.007). In univariate Cox regression, immunomodulator (HR 4.7, 95% CI 1.3–16.4, *p* = 0.02) and biologics use (HR 4.9, 95% CI 1.4–17.5, *p* = 0.01) were significant predictors of relapse, whereas disease extent showed only a non-significant trend with wide CIs. Baseline fecal calprotectin was higher in the relapse group (182 ± 370 μg/g vs. 108 ± 164 μg/g) but was not statistically significant. *Conclusions*: Approximately one-quarter of UC patients who achieved dual remission relapsed within 4 years. These findings highlight the limitations of using dual remission as the sole therapeutic endpoint and underscore the need for additional prognostic factors. High-risk subgroups—such as those with extensive disease or prior exposure to advanced therapies—may require closer monitoring and individualized strategies. Future multicenter studies integrating clinical, endoscopic, histologic, and biomarker data are needed to refine relapse prediction.

## 1. Introduction

Over the past decade, the therapeutic targets for ulcerative colitis (UC) have expanded beyond symptom control to include mucosal healing, driven by evidence linking endoscopic remission with favorable long-term outcomes, such as sustained remission, reduced hospitalization, and lower colectomy rates [1,2,3].

More recently, histological remission has emerged as an additional therapeutic target, based on studies that have revealed that microscopic inflammation may persist despite endoscopic quiescence and contribute to an increased risk of relapse and complications [4,5,6]. In line with these findings, the STRIDE-II consensus guidelines have endorsed histological remission as an adjunctive goal in UC management [2].

However, these guidelines also acknowledge several limitations: histological remission is difficult to achieve in routine clinical practice, lacks standardized scoring systems, and is subject to poor interobserver agreement [2,7,8]. Moreover, the incremental benefit of histological healing beyond endoscopic healing remains uncertain, and the number required to achieve a clinically meaningful advantage may be relatively high [2].

Notably, most of the current evidence originates from Western populations, with limited real-world data to address the long-term outcomes of histological remission in Asian cohorts. Therefore, we conducted a prospective observational study to investigate the long-term clinical outcomes and potential predictors of relapse in patients with UC who achieved both endoscopic and histological remission.

## 2. Materials and Methods

### 2.1. Study Design and Patients

This single-center, prospective observational study was conducted at St. Vincent’s Hospital, College of Medicine, The Catholic University of Korea. Consecutive adult outpatients with an established diagnosis of UC were prospectively enrolled between June 2014 and May 2018. Colonoscopy was performed either for colorectal cancer prevention or for routine disease monitoring, both of which are standard components of long-term UC care. The study was reviewed and approved by the Institutional Research Ethics Board of the hospital (IRB number:VC14OIMI0061). This study was conducted according to the principles of the Declaration of Helsinki. Written informed consent was obtained from all the participants.

Inclusion criteria were: (1) clinically inactive UC with no recent changes in medical therapy during the preceding year [9,10], (2) achievement of endoscopic remission defined as a Mayo endoscopic subscore (MES) of 0 [11], and (3) histologic remission defined as a Nancy histological index ≤1 [12]. All patients were required to undergo a minimum follow-up of 12 months. We reconfirmed that all included participants had MES = 0 at baseline, as documented in Supplementary Table S1, which lists each patient’s baseline MES (all = 0), Nancy index (≤1), and the date of index endoscopy/biopsy. For clarity, the term “Mayo score” in Table 1 refers to the clinical score, not the endoscopic subscore (MES).

Exclusion criteria were: (1) diagnosis of colorectal cancer; (2) concurrent infectious colitis; (3) signs or symptoms suggestive of intestinal obstruction; (4) pregnancy; and (5) incomplete clinical, endoscopic, or histologic data.

Demographic and clinical data were collected at baseline, including age, sex, disease duration, disease extent (classified as E1, E2, or E3 based on the Montreal classification), and medication history (e.g., use of immunomodulators or biologics). Laboratory parameters, including fecal calprotectin (FC) levels, were also recorded.

In Korea, UC is managed under the National Health Insurance system, which supports a conservative step-up therapy approach. Accordingly, 5-aminosalicylic acid (5-ASA) is generally used as first-line maintenance therapy, and escalation to immunomodulators (IMM) and/or biologics is considered only in cases of disease progression or inadequate response. Systemic corticosteroids may be used for moderate to severe UC but were not permitted as maintenance therapy. Biologic agents were reserved for patients with steroid-dependent or steroid-refractory disease, in accordance with Korean National Health Insurance treatment policies and national UC treatment guidelines [13].

### 2.2. Endoscopic and Histologic Assessment

Endoscopic evaluation was performed using a standard colonoscopy. The most severely affected area was scored according to the MES, and a score of 0 was used to define endoscopic remission [11].

Histologic remission [12] was assessed by experienced gastrointestinal pathologists using the Nancy index, blinded to clinical and endoscopic data. At least two biopsies were systematically obtained from healed mucosa in both the rectum and sigmoid colon, with additional samples taken from any endoscopically suspicious area at the discretion of the endoscopist [14]. The highest grade among all sampled sites (“worst-grade” rule) was used to classify histologic status. A Nancy index score of ≤1, indicating absence of acute inflammatory cell infiltrates, was considered consistent with histologic remission.

### 2.3. Follow-Up and Definition of Clinical Relapse

The patients were followed up at regular outpatient intervals for at least 12 months. Clinical relapse was defined as an increase in the total Mayo score by more than 3 points or the initiation of new treatment with systemic corticosteroids or biologics due to a disease flare.

### 2.4. Statistical Analysis

Descriptive data were presented as means ± standard deviation or medians with interquartile ranges, as appropriate. Kaplan–Meier survival curves were constructed to estimate cumulative relapse-free survival, and the number of patients at risk was displayed below each curve. Differences between survival curves were assessed using the log-rank test.

Risk factors associated with relapse were evaluated using univariate Cox proportional hazards regression analysis, and hazard ratios (HRs) with 95% confidence intervals (CIs) and *p*-values were calculated. Given the modest number of relapse events, multivariate Cox regression was not performed to avoid statistical instability and overfitting. Statistical significance was set at *p* < 0.05. All statistical analyses were performed using SPSS software (version 20, IBM Corp., Armonk, NY, USA).

## 3. Results

### 3.1. Patient Characteristics

A total of 41 patients with UC who achieved endoscopic and histological remission were prospectively enrolled. The mean age of the cohort was 54 ± 14 years, and 56.1% (n = 23) of the patients were male. The median disease duration was 55 months (range, 3–262 months), and the median follow-up period was 54 months (range, 17–78 months). At the time of inclusion, 87.8% (n = 36) of the patients had a Mayo clinical score of 0, while 12.2% (n = 5) had a score of 1.

Regarding the disease extent based on the Montreal classification, 36.6% (n = 15) of the patients had proctitis (E1), 36.6% (n = 15) had left-sided colitis (E2), and 26.8% (n = 11) had extensive colitis (E3). Most patients received 5-aminosalicylic acid maintenance therapy (97.6%), 22.0% (n = 9) received immunomodulators, and 12.2% (n = 5) received biological agents. Additionally, 7.3% (n = 3) were receiving topical corticosteroids (budesonide enemas) at enrollment; systemic corticosteroids were not used as maintenance therapy. Only one patient (2.4%) was not on any maintenance medication at the time of enrollment (Table 1).

### 3.2. Relapse Outcomes

**Figure 1:**
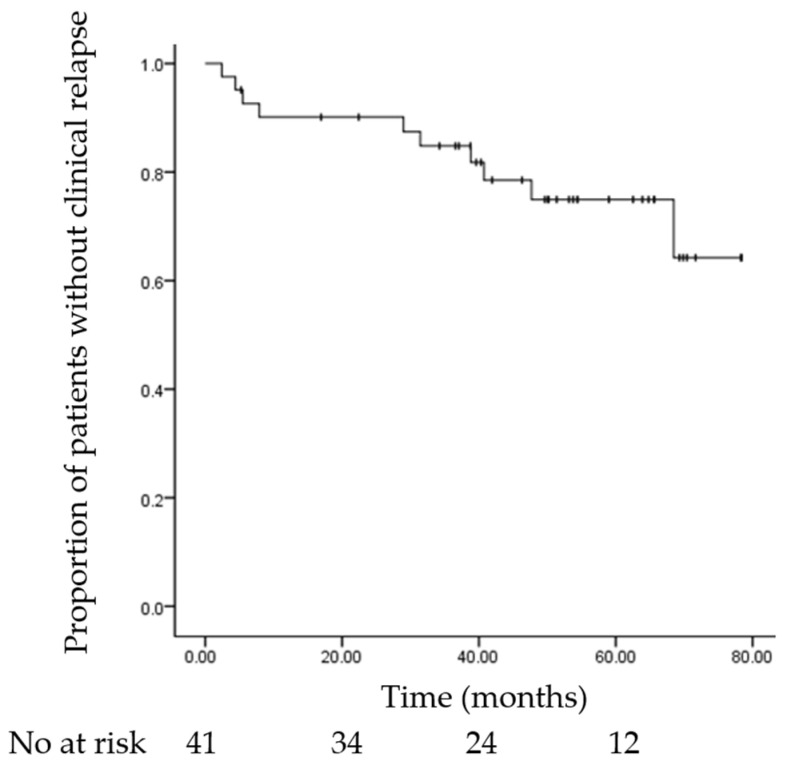
Kaplan–Meier curve of relapse-free survival for the overall cohort. The number of patients at risk is shown below the plot. Median relapse-free survival was not reached during follow-up.

During the follow-up period, 10 patients (24.4%) experienced clinical relapse. The cumulative relapse rates were 9.8%, 10.3%, 15.8%, and 24.1% at 12, 24, 36, and 48 months, respectively (Figure 1).

**Figure 2:**
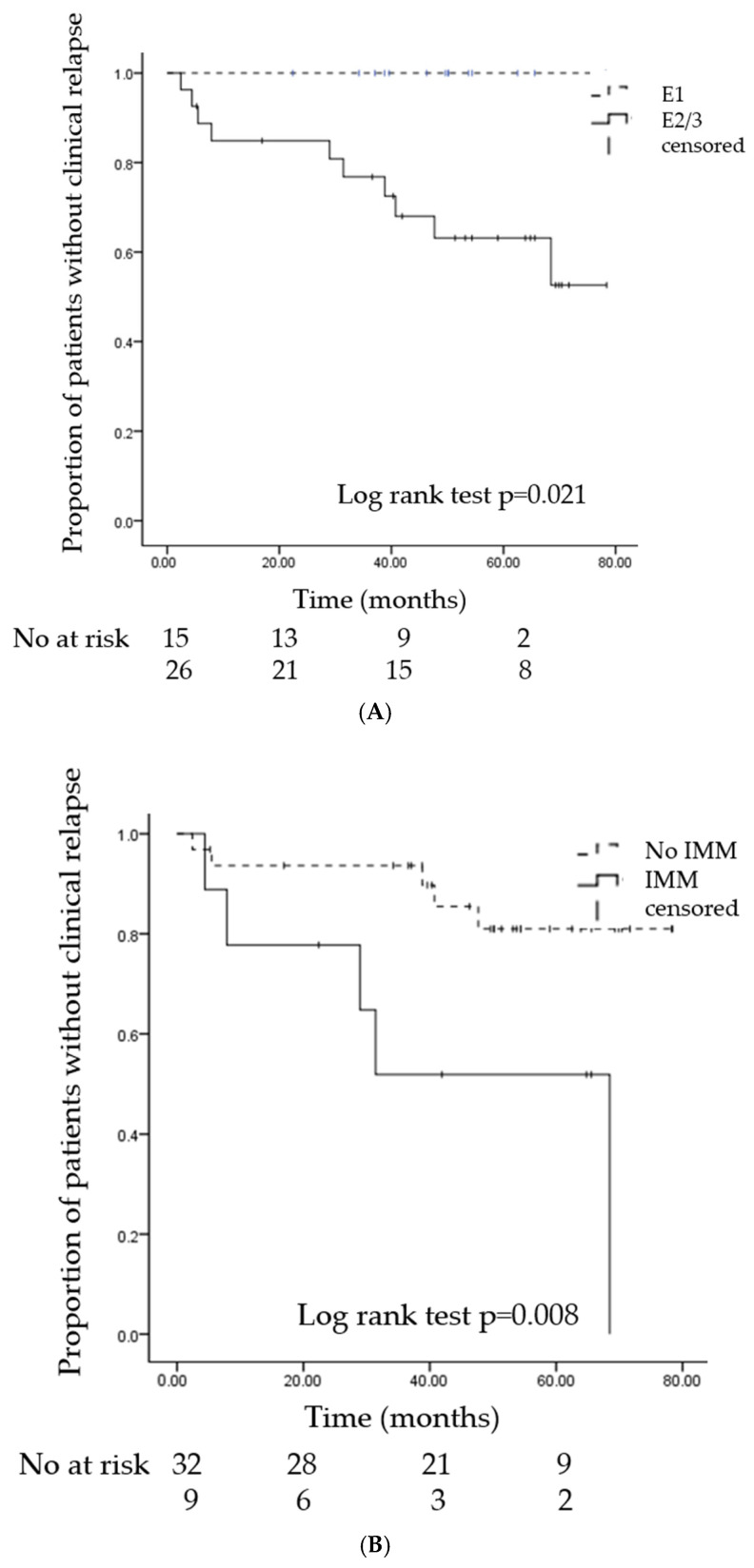

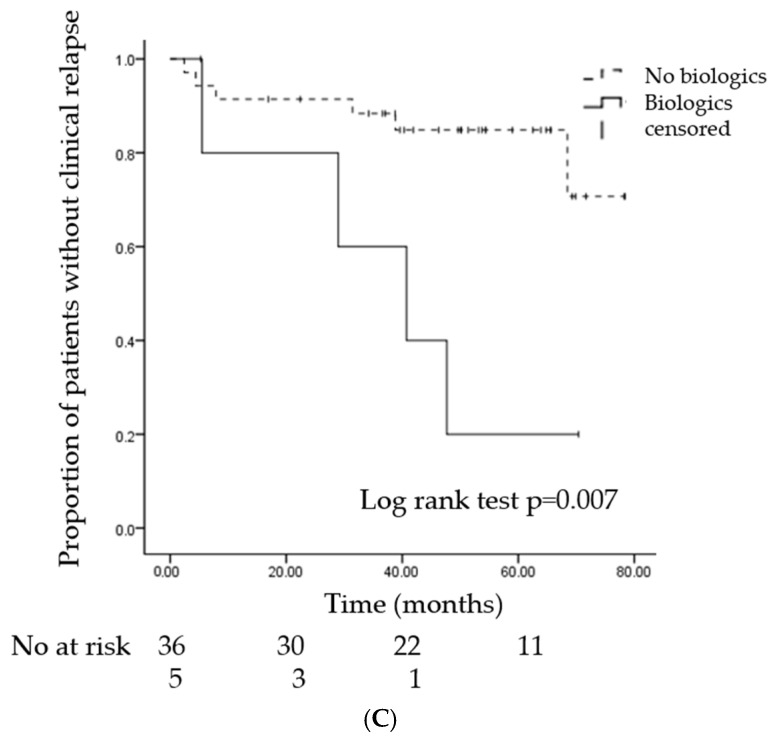
Kaplan–Meier curves of relapse-free survival by subgroup. (**A**) Patients with limited disease (E1, n = 15) versus non-E1 disease (E2 + E3, n = 26) (**B**) Patients receiving (n = 9) versus not receiving immunomodulators (IMM, n = 32) (**C**) Patients receiving (n = 5) versus not receiving biologics (n = 36). The number of patients at risk is shown below each plot, and Log-rank *p*-values are indicated within each panel. Median relapse-free survival was not reached in most subgroups.

When stratified by disease extent, patients with non-E1 disease (E2 + E3) showed a significantly higher risk of relapse compared with those with limited disease (E1) (log-rank *p* = 0.021; Figure 2A). Similarly, relapse occurred more frequently in patients receiving immunomodulators (log-rank *p* = 0.008; Figure 2B) and in those treated with biologics (log-rank *p* = 0.007; Figure 2C).

### 3.3. Risk Factors for Relapse

All relapses occurred among patients with non-E1 disease (E2 + E3; 10/26, 38.5%) versus none in E1 (0/15), *p* = 0.02), and the use of immunomodulators or biological agents at baseline was significantly associated with relapse. Among the patients who relapsed, 50% were on immunomodulators, compared with only 13% in the non-relapse group (*p* = 0.01), and 40% were on biologics, compared to only 3% in the non-relapse group (*p* = 0.02). The relapse group had a higher mean FC level (181.8 ± 370.1 μg/g) than the non-relapse group (108.4 ± 164.4 μg/g), although this difference did not reach statistical significance. Furthermore, no statistically significant differences were observed between the groups in terms of disease duration or CRP levels (Table 2A).

Univariate Cox regression analysis identified immunomodulator and biologics use as significant predictors of clinical relapse (Table 2B). Use of immunomodulators was associated with a 4.7-fold increased hazard of relapse (HR 4.7, 95% CI 1.3–16.4, *p* = 0.02), and biologics use was similarly associated with a higher relapse risk (HR 4.9, 95% CI 1.4–17.5, *p* = 0.01). Although patients with non-E1 disease (E2 + E3) tended to have a higher hazard (HR 41.1, 95% CI 0.08–9963.2), this finding did not reach statistical significance, likely due to the limited number of events. Disease duration, CRP, and FC were not significantly associated with relapse in this analysis.

## 4. Discussion

Our prospective study evaluated the long-term outcomes of patients with UC who achieved both endoscopic remission (MES 0) and histologic remission (Nancy histologic index ≤1), with a median follow-up of 55 months. Despite meeting these stringent dual remission criteria, approximately one-quarter (24.4%) of the patients experienced clinical relapse within four years. These findings highlight the potential limitations of relying solely on endoscopic and histological remission as definitive markers of durable remission in clinical practice.

Histological remission has been proposed as an adjunctive therapeutic target based on evidence that persistent microscopic inflammation, even in endoscopically normal mucosa, increases the risk of relapse and complications [6,7]. A meta-analysis published in 2020 included 757 UC patients in endoscopic remission (MES 0 or equivalent) [15]. The findings revealed that histological remission was associated with a 63% lower risk of clinical relapse than persistent histological activity (RR 0.37, 95% CI 0.24–0.56). Importantly, the protective effect of this target diminished over time, as relapse continued to accumulate during the extended follow-up period. The relative risk of relapse in patients with histologic remission compared to those with persistent histologic activity was significantly lower in studies with a follow-up longer than 12 months (RR 0.22) than in those with a shorter follow-up (RR 0.48; *p* < 0.01). Additionally, in patients with UC in endoscopic remission (MES 0), the estimated annual risk of clinical relapse in those who achieve histological remission was approximately 5.0%.

Furthermore, the absence of standardized histologic criteria, substantial inter-observer variability, and the lack of a universally accepted definition of “histologic remission” represent significant limitations to its widespread implementation in routine clinical practice [5,16].

There is a growing consensus that the current definition of disease remission, which is based primarily on clinical and endoscopic outcomes, should be expanded to include histological assessment within a multidimensional framework. This perspective is reflected in a recent international expert consensus led by the International Organization for the Study of Inflammatory Bowel Diseases, which further formalized the concept of disease clearance: the concurrent achievement of clinical, endoscopic, and histological remission [17]. While this represents a comprehensive therapeutic goal, the consensus also emphasizes that the additional prognostic value of disease clearance compared to histological remission alone has not yet been established. Overall, this underscores the need to consider prognostic factors beyond histology. Although our study was not designed to evaluate the specific duration of remission required for long-term disease control, accumulating evidence indicates that achieving and maintaining deep remission is associated with favorable outcomes in UC. However, the optimal length of sustained remission needed to ensure durable stability remains to be clarified in future longitudinal studies.

Although our study focused on patients achieving dual remission (endoscopic and histologic), this concept is closely related to the more comprehensive framework of disease clearance. In our cohort, the majority of patients were also in clinical remission at baseline, suggesting that our population partially overlapped with the definition of disease clearance. Nevertheless, despite meeting these stringent criteria, approximately one-quarter of patients relapsed over long-term follow-up. This observation underscores that even disease clearance, as currently defined, may not guarantee durable remission, and that additional prognostic factors—such as disease extent, prior therapy exposure, or biomarker integration—remain important. Recent multicenter data, including the study by D’Amico et al., have highlighted the prognostic value of early disease clearance, but further validation in larger, real-world Asian cohorts is needed to establish its incremental benefit beyond histologic remission alone [18].

In our cohort, patients with non-E1 disease (E2 + E3) and those who received immunomodulators or biologics at baseline had a higher risk of relapse. Kaplan–Meier analyses confirmed these associations, showing significantly higher relapse rates in patients with extensive colitis (log-rank *p* = 0.021), immunomodulator use (*p* = 0.008), and biologics use (*p* = 0.007). In the univariate Cox regression, immunomodulator and biologics use remained significantly associated with relapse, whereas extensive disease showed only a non-significant trend with a very wide CI, likely due to the small number of events. These findings suggest that patients with more aggressive or treatment-refractory disease phenotypes remain at risk of relapse despite histologic healing, underscoring the importance of tailored maintenance strategies and vigilant monitoring in these subgroups. In our cohort, 10 patients experienced relapse during follow-up. Most required escalation of maintenance therapy, such as the initiation or dose optimization of immunomodulators or biologics, and several regained remission after treatment adjustment. These observations illustrate the real-world clinical course following relapse, although detailed post-relapse management was not the primary focus of this study.

We also incorporated FC measurements, which showed higher baseline levels in the relapse group compared to the non-relapse group (182 ± 370 μg/g vs. 108 ± 164 μg/g). Although this difference was not statistically significant, the finding is consistent with a previous study that supports FC as a non-invasive biomarker for detecting residual inflammation and predicting relapse risk in quiescent UC [19]. Another limitation is that FC was assessed only at baseline, without longitudinal follow-up. This may have reduced our ability to capture its predictive dynamics over time. Future studies should evaluate serial FC trends to better determine its role in relapse prediction.

Our relapse definition was intentionally stringent, requiring a Mayo score increase >3 or treatment escalation, to ensure clinical relevance. However, this approach may have failed to capture milder disease flares, leading to potential underestimation of relapse frequency. Future studies should stratify relapse by severity and assess outcomes following relapse, including treatment response and long-term disease course.

In interpreting these associations, an important limitation is the potential for confounding by indication. Patients receiving immunomodulators or biologics at baseline inherently represent a subgroup with more extensive or refractory disease, which itself confers a higher relapse risk. Therefore, the observed association between advanced therapy use and relapse should be interpreted with caution, as it may reflect underlying disease severity rather than a direct causal effect of treatment. Given the small number of relapse events in our cohort, we did not attempt a multivariate analysis, as such modeling would be statistically unstable and risk overfitting. Future multicenter studies with larger sample sizes are needed to disentangle treatment effects from disease severity in predicting relapse.

The present study has noteworthy strengths that include: first, a prospective design in a real-world Asian cohort with extended follow-up, which addresses the paucity of data in this population; second, the use of strict remission definitions (MES 0 and Nancy ≤1), which minimizes heterogeneity; third, a long median follow-up of 55 months, which enables the assessment of both sustained remission and late relapse; and fourth, the integration of biomarker data alongside clinical and histologic measures, which provides a multidimensional perspective on relapse risk.

This study has several limitations that should be considered when interpreting our findings. First, the modest sample size, low number of relapse events, and single-center design reduce the statistical power of the analyses and limit the generalizability of our results. Second, the observational nature of the study prevents causal inference regarding the identified risk factors. In addition, our stringent definition of relapse—requiring a >3-point increase in the Mayo score or treatment escalation—may have excluded mild flares, potentially leading to a slight underestimation of the relapse rate. Third, although our findings provide incremental insights, the novelty of the results is constrained by the small cohort size, highlighting the need for larger, multicenter validation studies. Finally, incorporation of novel biomarkers may further refine relapse prediction, and future studies should integrate molecular, immunological, and microbiome-based markers alongside clinical, endoscopic, and histological assessments to enable more individualized risk stratification. In particular, these findings highlight the need for multidimensional biomarker integration—combining microbiome, metabolomic, and immunologic profiles within clinical and histologic frameworks—to further improve relapse prediction, as suggested by recent multi-omics studies [20]. While our sample size was modest and the study conducted at a single center, several aspects strengthen the novelty of our work. These include the prospective enrollment design, the application of stringent and clearly defined dual-remission criteria, the long follow-up duration, and the focus on an Asian population, which is underrepresented in prior studies. Together, these features provide incremental knowledge regarding the long-term prognosis of patients achieving dual remission, although our results require validation in larger, multicenter cohorts. These analyses should be regarded as exploratory and hypothesis-generating, and they require validation in larger, multicenter studies.

## 5. Conclusions

Even patients with UC who achieve both endoscopic and histological remission remain at a measurable risk of long-term clinical relapse. These findings highlight the limitations of using dual remission as the sole therapeutic endpoint and support the incorporation of additional clinical, biochemical, and histological parameters into long-term disease management. High-risk subgroups, such as those with extensive disease or prior exposure to advanced therapies, may benefit from closer surveillance and individualized maintenance strategies. Future research should focus on identifying novel prognostic markers, refining histological targets, and adopting a multidimensional approach that integrates clinical, endoscopic, histological, and biomarker data to enhance individualized risk stratification and improve long-term outcomes in patients with UC.

## Figures and Tables

**Table 1:** Baseline characteristics of patients in the study.

	Total (n = 41)
Male Sex, n (%)	23 (56.1)
Age, yr †	54 ± 14
Disease duration, months *	55 [3, 262]
Follow-up duration, months *	54 [17, 78]
Clinical activity, n (%)	
Mayo score 0	36 (87.8)
Mayo score 1	5 (12.2)
Extension, n(%)	
Proctitis (E1)	15 (36.6)
Left-sided (E2)	15 (36.6)
Extensive (E3)	11 (26.8)
Current treatment, n (%)	
No medication	1 (2.4)
5-ASA	40 (97.6)
Immunomodulators	9 (22)
Biologics	5 (12.2)
topical corticosteroids (budesonide enema)	3 (7.3)

†, Mean ± standard deviation; * Median [range]; n, number; yr, years.

**Table 2:** Risk factors for clinical relapse: (**A**) *t*-test and χ^2^-test; (**B**) univariate Cox regression analysis.

**(A)**	**Relapse (n = 10)**	**No Relapse (n = 31)**	***p*** **-Value**
Extension: E2 + E3, n (%)	10 (100)	16 (52)	0.02
Immunomodulators, n (%)	5 (50)	4 (13)	0.01
Biologics, n(%)	4 (40)	1 (3)	0.02
Disease duration, months *	66 [3, 204]	51 [3, 262]	NS
CRP, mg/dL †	0.06 ± 0.03	0.13 ± 0.14	NS
Fecal calprotectin, μg/g †	181.8 ± 370.1	108.4 ± 164.4	NS
**(B)**	**Hazard Ratio**	**95% Confidence Interval**	***p*** **-Value**
Extension: E2 + E3	41.1	0.08–9963.2 **	NS
Immunomodulators	4.7	1.3–16.4	0.02
Biologics	4.9	1.4–17.5	0.01
Disease duration	1.0	1.0–1.1	NS
CRP	0.001	0–152.0 *	NS
Fecal calprotectin	1.0	1.0–1.1	NS

†, Mean ± standard deviation; * Median [range]; n, number. ** Estimates marked with an asterisk are unstable due to sparse events or quasi-separation in the Cox model and should be interpreted with caution.

## Data Availability

The datasets generated and analyzed during the current study are not publicly available due to patient privacy restrictions but are available from the corresponding author on reasonable request.

## Supplementary Materials

The following supporting information can be downloaded at: https://www.mdpi.com/article/10.3390/medicina61111968/s1, Table S1. Baseline Endoscopic and Histologic Status of Included Patients.

## Abbreviations

The following abbreviations are used in this manuscript:

UC	Ulcerative colitis
MES	Mayo endoscopic subscore
FC	fecal calprotectin

## References

[1] Neurath M.F., Travis S.P. (2012). Mucosal healing in inflammatory bowel diseases: A systematic review. Gut.

[2] Turner D., Ricciuto A., Lewis A., D’Amico F., Dhaliwal J., Griffiths A.M., Bettenworth D., Sandborn W.J., Sands B.E., Reinisch W. (2021). STRIDE-II: An Update on the Selecting Therapeutic Targets in Inflammatory Bowel Disease (STRIDE) Initiative of the International Organization for the Study of IBD (IOIBD): Determining Therapeutic Goals for Treat-to-Target strategies in IBD. Gastroenterology.

[3] Colombel J.F., Rutgeerts P., Reinisch W., Esser D., Wang Y., Lang Y., Marano C.W., Strauss R., Oddens B.J., Feagan B.G. (2011). Early mucosal healing with infliximab is associated with improved long-term clinical outcomes in ulcerative colitis. Gastroenterology.

[4] Christensen B., Hanauer S.B., Erlich J., Kassim O., Gibson P.R., Turner J.R., Hart J., Rubin D.T. (2017). Histologic Normalization Occurs in Ulcerative Colitis and Is Associated with Improved Clinical Outcomes. Clin. Gastroenterol. Hepatol..

[5] Lemmens B., Arijs I., Van Assche G., Sagaert X., Geboes K., Ferrante M., Rutgeerts P., Vermeire S., De Hertogh G. (2013). Correlation between the endoscopic and histologic score in assessing the activity of ulcerative colitis. Inflamm. Bowel Dis..

[6] Bryant R.V., Burger D.C., Delo J., Walsh A.J., Thomas S., von Herbay A., Buchel O.C., White L., Brain O., Keshav S. (2016). Beyond endoscopic mucosal healing in UC: Histological remission better predicts corticosteroid use and hospitalisation over 6 years of follow-up. Gut.

[7] Peyrin-Biroulet L., Bressenot A., Kampman W. (2014). Histologic remission: The ultimate therapeutic goal in ulcerative colitis?. Clin. Gastroenterol. Hepatol..

[8] Pai R.K., Jairath V., Vande Casteele N., Rieder F., Parker C.E., Lauwers G.Y. (2018). The emerging role of histologic disease activity assessment in ulcerative colitis. Gastrointest. Endosc..

[9] Lee J.M., Lee K.M., Kang H.S., Koo J.S., Lee H.S., Jeong S.H., Kim J.H., Kim D.B. (2023). Oral Sulfate Solution Is as Effective as Polyethylene Glycol with Ascorbic Acid in a Split Method for Bowel Preparation in Patients with Inactive Ulcerative Colitis: A Randomized, Multicenter, and Single-Blind Clinical Trial. Gut Liver.

[10] Farrokhyar F., Marshall J.K., Easterbrook B., Irvine E.J. (2006). Functional gastrointestinal disorders and mood disorders in patients with inactive inflammatory bowel disease: Prevalence and impact on health. Inflamm. Bowel Dis..

[11] Sharara A.I., Malaeb M., Lenfant M., Ferrante M. (2022). Assessment of Endoscopic Disease Activity in Ulcerative Colitis: Is Simplicity the Ultimate Sophistication?. Inflamm. Intest. Dis..

[12] Marchal-Bressenot A., Salleron J., Boulagnon-Rombi C., Bastien C., Cahn V., Cadiot G., Diebold M.D., Danese S., Reinisch W., Schreiber S. (2017). Development and validation of the Nancy histological index for UC. Gut.

[13] Choi C.H., Moon W., Kim Y.S., Kim E.S., Lee B.I., Jung Y., Yoon Y.S., Lee H., Park D.I., Han D.S. (2017). Second Korean Guideline for the Management of Ulcerative Colitis. Korean J. Gastroenterol. Taehan Sohwagi Hakhoe Chi.

[14] Magro F., Langner C., Driessen A., Ensari A., Geboes K., Mantzaris G.J., Villanacci V., Becheanu G., Borralho Nunes P., Cathomas G. (2013). European consensus on the histopathology of inflammatory bowel disease. J. Crohn’s Colitis.

[15] Yoon H., Jangi S., Dulai P.S., Boland B.S., Prokop L.J., Jairath V., Feagan B.G., Sandborn W.J., Singh S. (2020). Incremental Benefit of Achieving Endoscopic and Histologic Remission in Patients with Ulcerative Colitis: A Systematic Review and Meta-Analysis. Gastroenterology.

[16] Guardiola J., Arajol C., Armuzzi A. (2017). Is histologic remission in ulcerative colitis ready for prime time?. Dig. Liver Dis..

[17] D’Amico F., Magro F., Siegmund B., Kobayashi T., Kotze P.G., Solitano V., Caron B., Al Awadhi S., Hart A., Jairath V. (2024). Disease Clearance as a New Outcome in Ulcerative Colitis: A Systematic Review and Expert Consensus. Inflamm. Bowel Dis..

[18] D’Amico F., Fiorino G., Solitano V., Massarini E., Guillo L., Allocca M., Furfaro F., Zilli A., Bonovas S., Magro F. (2022). Ulcerative colitis: Impact of early disease clearance on long-term outcomes—A multicenter cohort study. United Eur. Gastroenterol. J..

[19] Theede K., Holck S., Ibsen P., Kallemose T., Nordgaard-Lassen I., Nielsen A.M. (2016). Fecal Calprotectin Predicts Relapse and Histological Mucosal Healing in Ulcerative Colitis. Inflamm. Bowel Dis..

[20] Akiyama S., Nishijima S., Kojima Y., Kimura M., Ohsugi M., Ueki K., Mizokami M., Hattori M., Tsuchiya K., Uemura N. (2024). Multi-biome analysis identifies distinct gut microbial signatures and their crosstalk in ulcerative colitis and Crohn’s disease. Nat. Commun..

