# Supplementary material for: Long-Term Clinical Outcomes of Ulcerative Colitis with Concurrent Endoscopic and Histologic Remission
Source: Medicina (Kaunas). 2025 Nov 2;61(11):1968. doi: 10.3390/medicina61111968 (PMC12654765; doi:10.3390/medicina61111968)
Supplement: Supplementary file 1 [file medicina-61-01968-s001.zip › medicina-3856343-supplementary.pdf]

Table S1. Baseline Endoscopic and Histologic Status of Included Patients.

Patient ID	MES at Baseline	Nancy Index at Baseline	Date of Index Endoscopy/Biopsy
1	0	1	2017-02-09
2	0	0	2016-06-27
3	0	0	2016-12-14
4	0	1	2017-07-12
5	0	1	2014-07-03
6	0	1	2016-08-10
7	0	1	2016-11-09
8	0	0	2014-09-15
9	0	1	2015-08-10
10	0	1	2015-04-16
11	0	0	2018-02-07
12	0	1	2017-08-16
13	0	1	2017-11-13
14	0	1	2017-12-06
15	0	1	2017-01-12
16	0	1	2017-12-04
17	0	1	2015-06-29
18	0	1	2015-08-20
19	0	1	2015-07-23
20	0	1	2017-03-27
21	0	1	2016-07-04
22	0	1	2015-10-14
23	0	0	2016-07-25
24	0	1	2016-02-15
25	0	1	2015-07-27
26	0	1	2015-05-11
27	0	1	2015-05-18
28	0	1	2015-04-06
29	0	0	2017-09-14
30	0	1	2018-02-01
31	0	1	2016-03-23
32	0	1	2016-10-06
33	0	0	2016-12-21
34	0	1	2015-03-23

35	0	1	2016-05-16
36	0	0	2016-07-18
37	0	1	2016-08-18
38	0	1	2015-06-01
39	0	1	2015-07-02
40	0	1	2017-10-19
41	0	1	2017-06-12
